# Clinical and Electrocardiographic Characteristics in NSTEMI Patients With Acute Total Occlusion of Culprit Left Circumflex Artery

**DOI:** 10.1111/anec.70070

**Published:** 2025-04-03

**Authors:** Yongshi Wei, Doudou Pei, Jiang Deng, Bryan Richard Sasmita, Lijun Mao, Fengpeng Jia

**Affiliations:** ^1^ Department of Geratology Yongchuan Hospital of Chongqing Medical University Chongqing China; ^2^ Department of Cardiology Chongqing University Qianjiang Hospital Chongqing China; ^3^ Department of Cardiology Yongchuan Hospital of Chongqing Medical University Chongqing China; ^4^ Department of Cardiology The First Affiliated Hospital of Chongqing Medical University Chongqing China; ^5^ Department of Respiratory and Critical Care Medicine Jiangjin Second People's Hospital of Chongqing Chongqing China

**Keywords:** acute total occlusion, ECG alterations, left circumflex artery, non‐ST segment elevation myocardial infarction

## Abstract

**Background:**

Complete blockage of the culprit coronary artery is associated with 30% of NSTEMI (non‐ST‐segment elevation myocardial infarction) patients. The culprit vessel in the left circumflex artery (LCX) is more prevalent in this subset of individuals. These people's clinical features and ischemia alterations on electrocardiography (ECG) are unknown. The goals of this study were to examine clinical features and identify predicted ECG abnormalities in NSTEMI patients with complete blockage of the culprit LCX.

**Methods:**

This study enrolled 5215 consecutive NSTEMI patients' data. A total of 180 people were diagnosed with acute total occlusion of the culprit artery (ATOCA). Based on the culprit vessel, the patients were classified into three groups:ATOCA in the LAD (*n* = 46), ATOCA in the RCA (*n* = 38) and ATOCA in the LCX (*n* = 96). Furthermore, basic clinical data, ECG alterations, and the occurrence of major adverse cardiac events (MACEs) were gathered and examined.

**Results:**

In this single‐center investigation, we discovered that ATOCA was more prevalent in patients with NSTEMI in the LCX group. Patients with culprit LCX were more prone to having multivessel coronary disease (*p* = 0.015), poorer LVEF (*p* = 0.040), and a lower revascularization success rate (*p* = 0.019) during hospitalization, although there were no significant differences in MACEs in short and long follow‐up. STV5 + STV6 ≥ 2.5 mm (OR = 2.595, 95% CI: 1.297 ~ 5.192) and T‐wave imbalance (defined as an upright T‐wave in V1 with an amplitude larger than V6 (T1–T6 ≥ 1 mm) recorded from the P‐R interval)(OR = 3.871, 95% CI: 1.820 ~ 8.231) were shown to be independent predictors of NSTEMI patients with acute complete blockage of the culprit LCX in multivariate regression analysis.

**Conclusion:**

The LCX is the most prevalent culprit vessel with acute complete occlusion in NSTEMI patients, yet it has little effect on clinical outcomes. This subset of patients may be predicted by STV5 + STV6 ≥ 2.5 mm and T‐wave imbalance.

## Graphical abstract

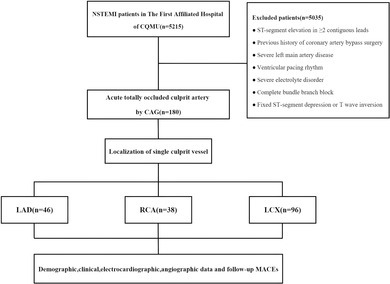

## 1 Introduction

The term “NSTE‐ACS” (non‐ST‐segment elevation acute coronary syndrome) refers to a series of clinical syndromes caused by the rupture or invasion of coronary atherosclerotic plaque and consequent partial occlusive thrombosis (Neumann et al. 2019). The electrocardiogram (ECG) frequently shows no ST segment elevation. Non‐ST elevation myocardial infarction (NSTEMI) is one of the most common kinds of NSTE‐ACS, with a growing incidence rate, particularly among younger people (Zhang et al. 2016; Li et al. 2014). Long‐term follow‐up is associated with a greater risk of mortality than STEMI (Pilgrim et al. 2016). NSTEMI has recently emerged as one of the leading causes of cardiovascular disease mortality, imposing a significant economic burden on the world (Naghavi et al. 2017).

Although the concept of acute myocardial infarction (AMI) is mostly based on an elevation in myocardial damage markers (Thygesen et al. 2019), ECG remains the quickest and easiest diagnostic technique in clinical practice. In contrast to STEMI, the infarct‐related artery (IRA) in most NSTEMI patients is not entirely blocked; therefore, the ECG mostly shows ST segment depression or/and T‐wave inversion (Amsterdam et al. 2014). It has been discovered that around one‐third of NSTEMI patients also have acute complete blockage of the culprit artery (Hung et al. 2018; Best et al. 2010), with the proportion of the culprit vessel in the left circumflex artery (LCX) reaching 60% (Best et al. 2010), while the ECG detective rate is around 32%–48% (Stribling et al. 2011). When compared to NSTEMI patients with partial blockage of the IRA, these patients have significantly larger myocardial infarction regions and greater rates of cardiovascular adverse events and mortality (Khan et al. 2017). Although early revascularization (12 h) in high‐risk patients might help improve prognosis (Yerasi and Weintraub 2021; Lawton et al. 2022), identifying NSTEMI patients with acute complete occlusive LCX by ECG alterations continues to be a major issue. Several investigations have postulated unique ECG abnormalities as possible predictors of IRA in patients with NSTEMI, including T‐wave imbalance (Smith et al. 2012), de‐Winter ST/T‐wave complex (Wall et al. 2018), N‐wave (Niu et al. 2013), and T2‐T6mm (Rovai et al. 2016). Their clinical values require additional confirmation due to their limited sensitivity and specificity. The objective of this research was to describe these patients' clinical features as well as to create sensitive and specific ECG indicators for identifying individuals with acute full LCX blockage without ST‐segment elevation.

## 2 Materials and Methods

### 2.1 Population

**FIGURE 1:**
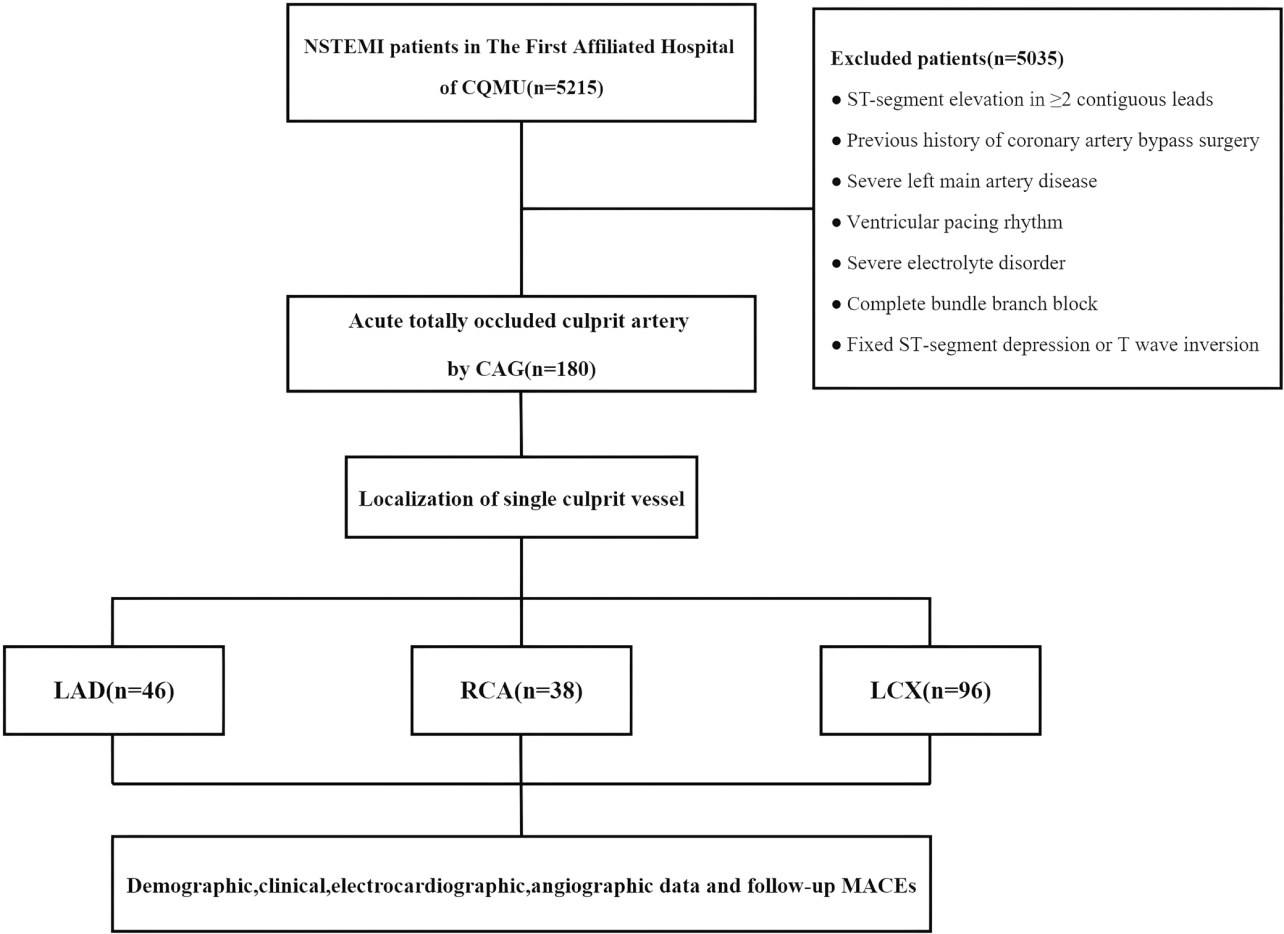
Flow chart showing patients selection, grouping and data analysis strategy.

Between January 2016 and May 2019, 5215 consecutive patients with NSTEMI were hospitalized at our facility, and 180 of them were evaluated to have acute total occlusion of the culprit artery (ATOCA) by coronary angiography and had percutaneous coronary intervention (PCI) treatment for a single IRA. The patients were separated into three groups based on the location of the single culprit vessel: ATOCA in the left anterior descending group (LAD group), ATOCA in the right coronary artery group (RCA group), and ATOCA in the left circumflex coronary artery group (LCX group). Basic clinical data, ECG results, coronary artery structural characteristics, medicinal therapy, the incidence of major adverse cardiac events (MACEs) during hospitalization, and one‐year follow‐up were collected and evaluated (Figure 1). The study protocol was approved by the Ethics Committee of “The First Affiliated Hospital of Chongqing Medical University”, and informed permission was obtained from all enrolled patients.

### 2.2 Inclusion Criteria

The following criteria for inclusion were used: (1) age of 18 years; (2) presence of acute chest pain for 20 min; (3) new ST‐segment depression 0.5 mm or negative T‐waves 2 mm in 2 contiguous leads; (4) serum troponin T levels with at least one value over the 99th percentile upper reference limit (Amsterdam et al. 2014; Collet et al. 2020); and (5) single acute complete blockage of the culprit artery verified by coronary angiography.

### 2.3 Exclusion Criteria and the Definition of Related Diagnosis

Patients with any of the following conditions were barred from participating: (1) persistent ST‐segment elevation in two consecutive leads; (2) prior history of coronary artery bypass surgery; (3) severe left main artery disease; (4) ventricular pacing rhythm; (5) severe electrolyte disorder; (6) full bundle branch block; and (7) fixed ST depression (STD) and/or T wave inversion.

The term “myocardial infarction” was defined using the current universal meaning (Thygesen et al. 2019). NSTEMI was defined as an increase in troponin readings over the 99th percentile and at least one of the following criteria: angina or angina equivalent, new ischemic ECG alterations (i.e., T wave inversion (TWI) and/or ST depression (STD)), or a new regional wall motion abnormality in a pattern compatible with an ischemic etiology (Amsterdam et al. 2014).

Recurred acute myocardial infarction, cerebral ischemic stroke, heart failure, and all‐cause mortality are considered major adverse cardiovascular events. The presence of local intraluminal thrombus and/or the absence of anterograde coronary blood flow established the culprit total occlusive coronary. Furthermore, the 12‐lead ECG and bedside echocardiogram results were compatible with coronary angiography. The flow grade was determined using the Thrombolysis in Myocardial Infarction (TIMI) trial criteria (Lawton et al. 2022). A stenosis of 70% in at least two main epicardial coronary arteries was defined as multivessel disease. Concurrent coronary angiography was performed by two experienced invasive cardiologists who were unrelated to the clinical trial.

### 2.4 Electrocardiography Definition

T‐wave imbalance (Smith et al. 2012) is defined as an upright T‐wave in V1 with an amplitude larger than V6 (T1–T6 ≥ 1 mm) recorded from the P‐R interval. The de‐Winter ST/T wave complex (Wall et al. 2018) consists of a 1 mm ST depression at the J‐point, followed by up‐sloping ST segments and peaked symmetrical T‐waves. N‐wave (Niu et al. 2013) is defined as (1) a notch or deviation in the terminal QRS complex and (2) a height of 2 mm measured from the PR segment. The difference in T‐wave amplitude between V2 and V6 ≥ 6 mm is described as T2‐T6 ≥ 6 mm (Rovai et al. 2016). The sum of the T‐wave amplitudes of L1 and V6 ≤ 0 mm is defined as TL1 + T6 ≤ 0 mm (Rovai et al. 2016). Two electrophysiological researchers, unaware of the coronary angiographic data, reviewed and analyzed all ECG findings. Differences were resolved by consensus or interpretation by a third researcher.

### 2.5 Statistical Analysis

Continues variables were expressed by mean ± SD. Independent sample T‐test was used between the two groups, and One‐Way ANOVA or Kruskal‐Wallis test was used between the three groups. Categorical variables were presented as numbers and percentages (%), analyzed by the Fisher exact test or chi‐square test. The electrocardiographic predictors of the culprit vessel in LCX were first investigated using univariate logistic regression analysis. Multiple logistic regression was used to identify the final independent predictors of key endpoints. Statistical significance was defined as a P value of 0.05. All calculations were performed with the SPSS (Version 23.0) program.

## 3 3‐Results

### 3.1 Baseline Characteristics

This single‐center study included 180 participants. Figure 1 depicts the patients' inclusion and exclusion criteria, the separated groups, and the data analysis approach. Patients were separated into three groups based on acute complete occlusion of the culprit vessel: ATOCA in the LAD, ATOCA in the RCA, and ATOCA in the LCX. The incidences of ATOCA in the LAD, RCA, and LCX groups were 25.6% (46 cases), 21.1% (38 cases), and 53.3% (96 cases), respectively. There were no variations in their baseline clinical features between the three groups (Table 1).

**TABLE 1:** Baseline characteristics of patients with NSTEMI.

Variable	LAD (*n* = 46)	RCA (*n* = 38)	LCX (*n* = 96)	*p*
Age (y)	62.6 ± 12.1	63.2 ± 13.4	64.8 ± 13.0	0.594
Gender, male (*n*, %)	27 (58.7)	26 (68.4)	68 (70.8)	0.348
BMI, kg/m^2^	24.3 ± 2.7	24.1 ± 3.3	24.9 ± 3.3	0.350
Smoking (*n*, %)	24 (52.2)	25 (65.8)	58 (60.4)	0.432
Prior PCI (*n*, %)	1 (2.2)	0	6 (6.3)	0.266
Prior stroke (*n*, %)	2 (4.3)	1 (2.6)	7 (7.3)	0.686
Hypertension (*n*, %)	27 (58.7)	22 (57.9)	59 (61.5)	0.910
Diabetes mellitus (*n*, %)	19 (41.3)	17 (44.7)	33 (34.4)	0.480
Hypercholesterolemia (*n*, %)	27 (58.7)	24 (63.2)	56 (58.3)	0.871
Peripheral vascular disease (*n*, %)	9 (19.6)	6 (15.8)	8 (8.3)	0.145
Renal disease (*n*, %)	4 (8.7)	2 (5.3)	4 (4.2)	0.518

Abbreviations: BMI:body mass index; PCI:percutaneous coronary intervention.

### 3.2 Clinical Findings

Table 2 shows that cardiac troponin (*p* < 0.001), brain natriuretic peptide (*p* < 0.001), and left ventricular end‐diastolic width (*p* = 0.003) were substantially greater in the LCX group, whereas LVEF (*p* = 0.040) was lower in the other two groups (Table 2). Furthermore, across the three groups, patients with culprit LCX had the greatest frequency of moderate or severe mitral regurgitation (*p* = 0.026). Other clinical examinations, however, revealed no differences between the three groups.

**TABLE 2:** Clinical findings in patients with NSTEMI.

Variable	LAD (*n* = 46)	RCA (*n* = 38)	LCX (*n* = 96)	*p*
WBC × 10^9^/L	8.7 ± 3.3	8.7 ± 2.5	9.1 ± 3.2	0.607
Hemoglobin, g/L	139.9 ± 19.3	134.2 ± 16.8	132.1 ± 21.7	0.099
Platelet × 10^9^/L	211.9 ± 60.4	199.9 ± 52.2	207.5 ± 65.7	0.669
Cholesterol, mmol/L	4.4 ± 1.2	4.4 ± 1.0	4.2 ± 1.0	0.401
Low density lipoprotein, mmol/L	2.9 ± 1.2	2.8 ± 0.9	2.7 ± 0.9	0.316
Alanine transaminase, U/L	38.9 ± 32.7	35.9 ± 25.8	36.9 ± 18.2	0.838
Aspartate amino transferase, U/L	48.9 ± 47.6	48.4 ± 42.4	51.7 ± 30.9	0.874
Creatinine, μmol/L	79.1 ± 29.3	86.6 ± 28.8	86.2 ± 46.1	0.564
Uric acid, μmol/L	335.4 ± 142.7	366.4 ± 106.9	334.0 ± 93.2	0.288
BNP, pg/ml	50.8 (22.1256.3)	133.5 (67.7284.5)	218.5 (103,534)	**0.000**
Admission troponin, pg/ml	0.4 (0.2,1.7)	0.6 (0.1,4.0)	2.3 (0.4,7.6)	**0.000**
Serum potassium, mmol/L	4.1 ± 0.4	4.1 ± 0.4	4.0 ± 0.4	0.213
Sinus bradycardia (*n*, %)	7 (15.2)	8 (21.1)	11 (11.5)	0.357
Atrial fibrillation (*n*, %)	2 (4.3)	1 (2.6)	4 (4.2)	1.000
Atrioventricular block (*n*, %)	7 (15.2)	5 (13.2)	17 (17.7)	0.797
LVDD, mm	48.0 ± 4.9	48.0 ± 4.9	50.5 ± 4.8	**0.003**
LVEF, %	59.0 ± 6.0	59.0 ± 5.8	56.6 ± 6.8	**0.040**
Killip classification > 3 (*n*, %)	1 (2.2)	1 (2.6)	7 (7.3)	0.475
Moderate–severe MR (*n*, %)	1 (2.2)	3 (7.9)	16 (16.7)	**0.026**

*Note:* Significant *p* values are in boldface.

Abbreviations: BNP:B‐type natriuretic peptide; LVDD:left ventricular end‐diastolic diameter; LVEF:left ventricular ejection fraction; MR: mitral regurgitation; WBC:white blood cell.

### 3.3 Anatomical Characteristics of Coronary Artery Lesions and the Results of PCI

The three groups had a comparable proportion of dominant coronary arteries (*p* = 0.076). The occlusive location of the culprit coronary arteries did not differ across the three groups (*p* = 0.063). Patients in the LCX group were more likely to have multiple vascular lesions (*p* = 0.015) and a larger proportion of collateral circulation (*p* = 0.022) than those in the other two groups. The success rate of revascularization in the LCX group was considerably lower (*p* = 0.019) when compared to the LAD and RCA groups (Table 3).

**TABLE 3:** Coronary angiography anatomic findings and PCI treatments.

Variable	LAD (*n* = 46)	RCA (*n* = 38)	LCX (*n* = 96)	*p*
Number of diseases vessels (*n*, %)				**0.015**
Single vessel	40 (87.0)	33 (86.8)	66 (68.8)	
Multi vessel	6 (13.0)	5 (13.2)	30 (31.3)	
Occlusive site (*n*, %)				0.063
Proximal lesion	30 (65.2)	15 (39.5)	51 (53.1)	
Middle‐distal lesion	16 (34.8)	23 (60.5)	45 (46.9)	
Coronary artery dominance (*n*, %)				0.076
Left	1 (2.2)	6 (15.8)	16 (16.7)	
Right	42 (91.3)	29 (76.3)	70 (72.9)	
Codominant	3 (6.5)	3 (7.9)	10 (10.4)	
Coronary collateral circulation (*n*, %)	3 (6.5)	7 (18.4)	25 (26.0)	**0.022**
FMC, h	48 (10.1102)	48 (11.3168)	48 (9.1144)	0.990
DTB, < 90 min (*n*, %)	4 (8.7)	5 (13.2)	9 (9.4)	0.797
PCI/PTCA procedure success (*n*, %)	44 (97.8)	37 (97.4)	82 (85.4)	**0.019**

*Note:* Significant *p* values are in boldface.

Abbreviations: DTB:door to Balloon time; FMC:first medical contact; PCI:percutaneous coronary intervention; PTCA:percutaneous transluminal coronary angioplasty.

### 3.4 Comparison of MACEs During Hospitalization and One Year Follow‐Up

**FIGURE 2:**
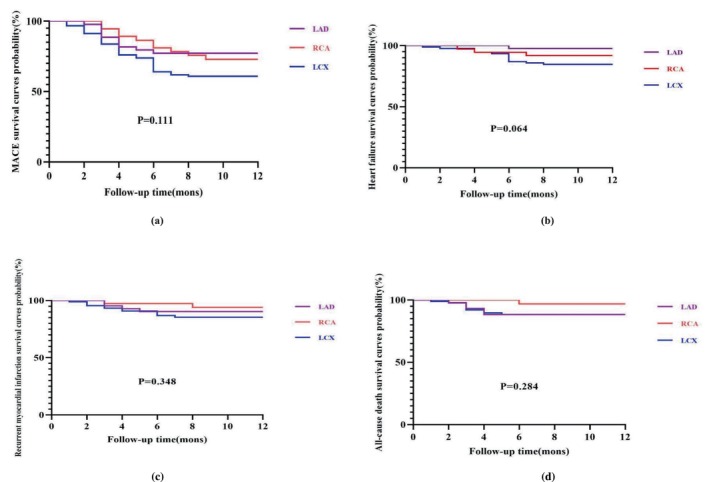
The MACEs Kaplan–Meier survival curves of patients with NSTEMI for 12 months follow up. There were no significant differences in all MACEs (*p* = 0.111; a), heart failure (*p* = 0.064;b), recurrent myocardial infarction (*p* = 0.348;c) and all‐cause death (*p* = 0.284;d). LAD, left anterior decending; LCX, left circumflex;MACE, major adverse cardiac events; RCA, right coronary artery.

All patients got the same medication therapy that was indicated by applicable recommendations (Table 4). There were no significant differences in the incidence rates of MACEs (heart failure, recurrent MI, stent thrombosis, all‐cause mortality, cerebral ischemic stroke, and cardiogenic shock) between the three groups during hospitalization and one‐year follow‐up (Table 4, Figure 2).

**TABLE 4:** Drug therapy and MACEs in hospital and 12 months follow‐up.

Variable	LAD (*n* = 46)	RCA (*n* = 38)	LCX (*n* = 96)	*p*
Drug therapy (*n*, %)				
Aspirin	42 (91.3)	34 (89.5)	90 (93.8)	0.615
Clopidogrel	25 (54.3)	17 (44.7)	46 (47.9)	0.655
Ticagrelor	21 (45.7)	21 (55.3)	50 (52.1)	0.655
Atorvastatin	35 (76.1)	31 (81.6)	76 (79.2)	0.824
Rosuvastatin	8 (17.4)	7 (18.4)	20 (20.8)	0.875
ACEI/ARB	38 (82.6)	31 (81.6)	70 (72.9)	0.336
β‐Blocker	39 (84.8)	29 (76.3)	79 (82.3)	0.592
In‐hospital MACEs (*n*, %)	7 (15.2)	4 (10.5)	16 (16.6)	0.668
Heart failure	6 (13.0)	4 (10.5)	15 (15.6)	0.730
Recurrent MI	1 (2.2)	4 (10.5)	6 (6.3)	0.257
CIS	0	0	0	1.000
All‐cause death	1 (2.2)	0	0	0.467
12‐months MACEs (*n*, %)				
Heart failure	1 (2.3)	3 (8.1)	14 (15.2)	0.061
Recurrent MI	4 (9.1)	2 (5.4)	12 (13.0)	0.477
CIS	0	0	0	1.000
All‐cause death	5 (11.4)	1 (2.7)	10 (10.9)	0.318

*Note:* At the 12‐months follow‐up,1 case was lost in LAD group,1 cases in RCA group and 4 cases in LCX group. Patients who died in hospital were not included in follow‐up.

Abbreviations: ACEI:angiotensin converting enzyme inhibitors; ARB:angiotensin receptor blocker; CIS: cerebral ischemic stroke; MACEs: major adverse cardiac events;MI:myocardial infarction.

### 3.5 Analysis of ECG Findings on Admission

In the majority of typical ECG alterations, no significant differences were seen (Table 5). Patients with culprit LCX vessels had more lateral Q‐waves or QS waves (*p* = 0.032), ST depression in V5‐V6 leads (*p* = 0.012), and T‐wave low‐flat or inversion in I/aVL and/or V5‐V6 (*p* = 0.014) than patients with culprit LAD or RCA. Furthermore, STV5 + STV6 ≥ 2.5 mm (The sum of the ST depressions of V5 and V6 leads ≥ 2.5 mm)was the most prevalent in participants with LCX (46.9 vs. 21.7 vs. 26.3%; *p* = 0.005). T‐wave imbalance was the most common of the hypothesized new ischemia ECG abnormalities in the LCX group when compared to the LAD and RCA groups (49 vs. 8.7 vs. 23.7%; *p* < 0.001).

**TABLE 5:** Electrocardiographic characteristics of the study patients.

Variable	LAD (*n* = 46)	RCA (*n* = 38)	LCX (*n* = 96)	*p*
Depolarization abnormalities (*n*, %)				
Anterior Q‐waves or QS waves	3 (6.5)	1 (2.6)	5 (5.2)	0.739
Lateral Q‐waves or QS waves	1 (2.2)	1 (2.6)	13 (13.5)	**0.032**
Inferior Q‐waves or QS waves	3 (6.5)	13 (34.2)	13 (13.5)	**0.002**
Prominent R‐wave in V1	1 (2.2)	0	7 (7.3)	0.212
Poor R‐wave progression in. V1‐V3	7 (15.2)	1 (2.6)	9 (9.4)	0.158
Repolarization abnormalities (*n*, %)				
ST depression in leads				
I, aVL	2 (4.3)	5 (13.2)	15 (15.6)	0.136
II, III, aVF	5 (10.9)	12 (31.6)	13 (13.5)	**0.020**
V1–V4	6 (13.0)	2 (5.3)	15 (15.6)	0.277
V5–V6	12 (26.1)	15 (39.5)	50 (52.1)	**0.012**
ST_V5_ + ST_V6_ ≥ 2.5 mm	10 (21.7)	10 (26.3)	45 (46.9)	**0.005**
T‐wave low‐flat or inversion in				
I, aVL	18 (39.1)	11 (28.9)	53 (55.2)	**0.014**
II, III, aVF	10 (21.7)	18 (47.4)	31 (32.3)	**0.044**
V1–V4	16 (34.8)	6 (15.8)	7 (7.3)	**0.000**
V5–V6	20 (36.4)	13 (27.7)	55 (50.9)	**0.016**
T‐wave imbalance	4 (8.7)	9 (23.7)	47 (49.0)	**0.000**
de‐Winter ST/T‐wave complex	1 (2.2)	2 (5.3)	3 (3.1)	0.737
T‐wave quantification				
T2‐T6 index, mm	−0.9 ± 7.5	6.1 ± 6.7	7.0 ± 6.9	**0.000**
T2‐T6 ≥ 6 mm (*n*, %)	11 (23.9)	22 (57.9)	58 (60.4)	**0.000**
TLI + T6 index, mm	5 (0, 7.0)	4.0 (−0.6,6.1)	1.0 (−1.0, 5.0)	**0.034**
TLI + T6 ≤ 0 mm (*n*, %)	15 (32.6)	11 (28.9)	45 (46.9)	0.088
N‐wave in leads (*n*, %)				
I and/or aVL	0	0	4 (4.2)	0.327
II and/or III and/or aVF	1 (2.2)	0	1 (1.0)	0.717

*Note:* ST_V5_ + ST_V6_ ≥ 2.5 mm, the sum of ST amplitude of V5 and V6 ≥ 2.5 mm;T2‐T6 ≥ 6 mm, the difference of T‐wave amplitude between V2 and V6 ≥ 6 mm;TL1 + T6 ≤ 0 mm, the sum of T‐wave amplitude of L1 and V6 ≤ 0 mm. Significant P values are in boldface.

There were few patients with De‐winter's ST/T and N‐wave, and there were no differences between the three groups (*p* > 0.05). The incidence of T2‐T6 ≥ 6 mm was comparable and considerably greater in the LCX and RCA groups than in the LAD group (60.4 vs. 57.9 vs. 23.9%, *p* < 0.001). Table 5 shows that there was no difference in TL1 + T6 ≤ 0 mm between the three groups (*p* = 0.088).

### 3.6 ECG Patterns Predicting Acute Total Occlusion of Culprit LCX in Patients With NSTEMI

**FIGURE 3:**
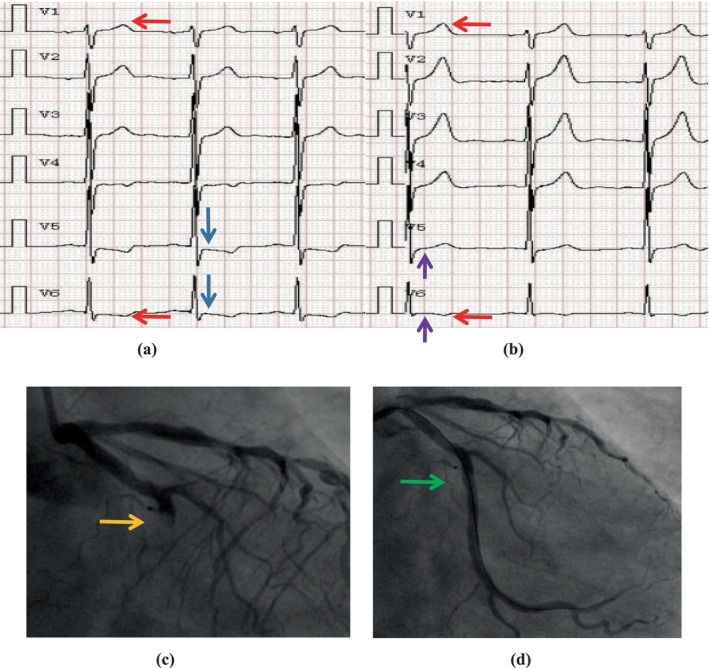
Representative electrocardiograms and angiograms of a NSTEMI patient with culpritLCX. The T wave is positive inV1, negativee inV6, which is defined as T‐wave imbalance (red arrow, a). In this patient, the ST segment is depressed in V5 and V6, thus STV5 + STV6 ≥ 2.5 mm (blue arrow, a). Angiograms demonstrate an acute total occlusion of LCX (yellow arrow, c) and re‐opened LCX artery (green arrow, d). After successful revascularization, ST‐depression gets better in V5 and V6 (purple arrow, b), but T‐wave imbalance is still present (red arrow, b).

Some ECG signs were related to LCX blockage, according to a univariate regression analysis (Table S1). STV5 + STV6 ≥ 2.5 mm and T‐wave imbalance were found to be independent predictors of ATOCA in the LCX by multivariate regression analysis (typical ECG ischemic alterations are given in Figure 3). In the study patients, the specificity, positive predictive value, and negative predictive value of STV5 + STV6 ≥ 2.5 mm in predicting culprit LCX were 76.2%, 69.2%, and 55.7%, respectively. T‐wave imbalance, on the other hand, showed specificity and positive and negative predictive values for identifying the culprit LCX of 84.5%, 78.3%, and 59.2%, respectively. Unfortunately, the STV5 + STV6 ≥ 2.5 mm and T‐wave imbalance showed 46.9 and 49.0% sensitivity for identifying the culprit LCX, respectively (Table 6).

**TABLE 6:** Value of ST_V5_ + ST_V6_ ≥ 2.5 mm and T‐wave imbalance in predicting culprit LCX in patients with NSTEMI.

	ST_V5_ + ST_V6_ ≥ 2.5 mm	T‐wave imbalance
True positive, *n*	45	47
True negative, *n*	64	71
False‐positive, *n*	20	13
False‐negative, *n*	51	49
Sensitivity, %	46.9 (36.7–57.3)	49.0 (38.7–59.3)
Specificity, %	76.2 (65.4–84.5)	84.5 (74.6–91.2)
PPV, %	69.2 (56.4–79.8)	78.3 (65.5–87.5)
NPV, %	55.7 (46.1–64.8)	59.2 (49.8–67.9)
Accuracy, %	23	33.4
LR (+)	1.97 (1.27–3.05)	3.16 (1.84–5.43)
LR (−)	0.70 (0.57–0.85)	0.60 (0.49–0.74)
Diagnostic odds ratio	2.82 (1.49–5.37)	5.24 (2.57–10.70)
Youden index (J)	0.23 (0.02–0.42)	0.33 (0.13–0.50)

Abbreviations: LR (+):positive likelihood ratio; LR (−):negative likelihood ratio; PPV:positive predictive value; NPV:negative predictive value.

## 4 Discussion

The primary findings of this study indicate that the left circumflex artery (LCX) is the most frequently identified culprit for completely occluded vessels in patients with non‐ST elevation myocardial infarction (NSTEMI), particularly in the presence of multivessel disease. Additionally, these patients exhibited a lower success rate of revascularization. Although patients with LCX as the culprit vessel demonstrated significantly elevated troponin levels, impaired cardiac function, and a higher prevalence of severe mitral regurgitation (MR) compared to those with other coronary artery involvement, the incidence of major adverse cardiac events (MACEs) was similar across the three groups at both short‐ and one‐year follow‐up. Furthermore, STV5 + STV6 ≥ 2.5 mm and T‐wave imbalance were identified as independent predictors of the LCX being the culprit vessel.

The current investigation found that the LCX was the most prevalent occlusive culprit vessel (53.3%) in NSTEMI patients, which was comparable with Pride et al. (2010) (48.4%). According to a systematic study and meta‐analysis, 17,212 (34%) of 60,898 individuals with NSTEMI had an occlusive coronary artery (OCA). The LCX was more likely to be the culprit artery in patients with OCA (Hung et al. 2018). Registry research discovered that acute occlusive LCX was the most common culprit lesion in 16,209 NSTEMI patients (Terlecki et al. 2021). The most prevalent acute occluded culprit artery among patients with NSTEMI was the left circumflex artery, according to the Polish National Registry (131,729 individuals) (35.86% NSTEMI with an occluded culprit artery versus 14.09% NSTEMI without an occluded culprit artery) (Terlecki et al. 2021).

Second, the varying patterns of the ST‐segment are connected to the occlusive position of the culprit LCX and the kind of dominant coronary artery. Because of its smaller size and/or more distant involvement as a culprit artery, the LCX is more likely to generate a lack of transmurality and a tiny infarcted region, which are frequently seen on the ECG as NSTEMI.

Third, because coronary occlusions can be dynamic, the ECG alterations will be sensitive to the spontaneous clearance of the occlusive thrombus in the culprit artery, which leads to the potential that the ST segment is temporary and commonly overlooked on non‐continuous 12‐lead ECGs, and so the initial ECG only shows ST segment depression or T wave inversion (Dzikowicz and Carey 2023). Furthermore, collateral circulation of the coronary artery will influence the existence of ST alterations in LCX with an acute complete blockage. Figueras et al. (2018) discovered that NSTEMI patients with occluded arteries had more collateral than NSTEMI patients without occluded arteries or STEMI patients. Ayad et al. (2021) discovered that NSTEMI patients who had an occluded artery had considerably more collateral than those who did not have an occluded culprit vascular. The depth and region of subendocardial ischemia vary with the abundance of collateral circulation, affecting the amount and extent of ionic current disruption (Klabunde 2017). The culprit LCX in our investigation was more likely to have proximal occlusion, multi‐vessel disorders, and existing collateral circulation. Some of the findings differ from those of other research. The disparity in ECG alterations in individuals with culprit LCX may vary based on a number of interconnected parameters such as occlusive position, implicated territory, transmural infarction, and collateral circulation (Gifft et al. 2020).

The clinical significance of abrupt, complete blockage of the culprit LCX in NSTEMI is still debated. Some studies show that patients with the occlusive culprit LCX had poorer results than those with non‐occlusive vessels in NSTEMI. Patients with an occlusive culprit artery had a poorer left ventricular ejection fraction, higher peak enzyme levels, and were at a greater risk of cardiogenic shock. In short or long follow‐up, there is an increase in mortality and recurrent myocardial infarction (Hung et al. 2018). A meta‐analysis of 40,777 patients with NSTEM found that 3440 (8.4%) of those with an acute culprit artery in the LCX had an elevated risk of MACE and all‐cause mortality (Khan et al. 2017). However, several studies have revealed that there is no substantial difference in short or long‐term results in this subset of patients, regardless of whether they have a culprit occlusive coronary artery or not (Kos et al. 2021). Ayad et al. (2021) also hypothesized that the existence of an occluded culprit artery does not significantly alter clinical outcomes for NSTEMI patients in‐hospital or after 6 months of follow‐up, but it is linked with a considerably greater prevalence of in‐hospital arrhythmia.

In this study, we looked at the influence of various occlusive culprit coronary arteries on both short‐ and long‐term outcomes. The findings revealed that the three groups had similar rates of MACEs in the hospital and after a year of follow‐up. Although peak troponin elevation was considerably greater in LCX culprit arteries than in RCA or LAD culprit vessels, LCX culprit vessels were not linked with poorer 30‐day or 1‐year outcomes in adjusted models (Halim et al. 2016). These disparities are mostly due to the different anatomical characteristics of the culprit vascular system and the duration between chest discomfort and first medical contact (FMC). Terlecki et al. discovered that the fraction of NSTEMI patients with poor outcomes has the longest time from pain to FMC and the lowest incidence of TIMI flow grade 3 following PCI (Terlecki et al. 2021). Despite having a comparable time to FMC, the culprit, LCX, had the greatest frequency of no‐reflow events after immediate PCI in our investigation. The low success rate was most likely related to delayed vascularization, convoluted arteries, and a larger burden of thromboses, which might result in elevated troponin, reduced cardiac function, and a higher frequency of severe MR in the ICU. Surprisingly, the worst indicators had no effect on in‐hospital or one‐year outcomes. Another possible explanation is that the results are connected not only to the occlusive culprit artery but also to the baseline comorbidities (Fernando et al. 2017).

It is well known that, unlike patients with STEMI, the electrocardiographic recognition of the acute occlusive culprit LCX in patients with NSTEMI remains a challenge. ST‐segment depression is usually found in V4–V6 leads in patients with NSTEMI, regardless of coronary artery anatomy (Tuohinen et al. 2018). Tan et al. (2013) suggested that ECG ST‐segment depression at admission is an independent predictor of in‐hospital mortality for NSTEMI patients. In the present study, we found that patients with acute occlusive culprit LCX are more frequently manifested as marked ST segment depression in V5–V6 leads, while the specific ECG quantitative index ST_V5_ + ST_V6_ ≥ 2.5 mm was an independent predictor (sensitivity was 46.9% and specificity was 76.2%). Wall et al. (2018) proposed that T‐wave imbalance is the most common new ischemic change in NSTEMI patients. The conclusion was consistent with our findings. We further demonstrated that it is more common in culprit LCX and presents as another independent predictor of total occlusion in culprit LCX (sensitivity is 49.0% and specificity is 84.5%). The mechanism of this ECG change may be related to normal lead V1, which presents as a negative T‐wave. When LCX is in acute complete occlusion, the lateral and posterior myocardia are ischemic and injured, and T‐wave repolarization of lead V1 is abnormal. The direction of the T‐wave vector is more advanced, that is, upright T‐wave, and the discrepancy between the T‐wave amplitudes of lead V1 and V6 is significantly increased.

T‐wave dynamic changes are also common in patients with NSTEMI. Rovai et al. (2016) described an abnormal T‐wave vector with anterior and inferior displacement in patients with NSTEMI, represented by the decreased T‐wave amplitude of lead I and V6 and the increased T‐wave amplitude of lead V2. Therefore, two indexes, T2‐T6 ≥ 6 mm and TL1 + T6 ≤ 0 mm, were both related to previous lateral myocardial infarction and LCX occlusion. In this study, multivariate analysis showed that both T2‐T6 ≥ 6 mm and TL1 + T6 ≤ 0 mm were not associated with the culprit LCX.

The de‐Winter ST/T‐wave complex was initially described as an ECG sign of proximal LAD occlusion. Wall et al. (2018) reported that this ECG change was found in 14% of NSTEMI patients and was more common in culprit LCX (37.5%). However, only 3.7% of patients with culprit LCX presented as de‐Winter ST/T‐wave in our study, which was similar to the conclusion of Pawel (3.9%) (Rostoff et al. 2020). Niu et al. (2013) found the N‐wave of the ECG ischemic pattern was in up to 77% of patients with NSTEMI, and patients with the N‐wave had a higher incidence of acute occlusive LCX, more MACEs in the hospital, and a longer follow‐up than patients without the N‐wave. Their clinical prognosis was similar to that of patients with STEMI, which could be regarded as “STEMI equivalents” (Yang et al. 2020). Rostoff et al. (2020) further pointed out that the N‐wave in aVL lead could be seen as an independent predictor of the occlusive culprit LCX. In our study, the occurrence of N‐wave was a very rare (4.3%) finding and was lower than the findings of Yang et al. (2020) (8.6%) and Niu et al. (2013) (77%). This great discrepancy is probably due to the different, complex pathophysiologies of participants and differences in the methodology of these studies.

## 5 5‐Limitations

### 5.1 There Were Some Limitations to This Study

- Firstly, our study was a single‐center retrospective study with a relatively small sample size, which may be affected by selection bias.
- Secondly, due to the lack of 18‐lead ECG data on admission, the posterior wall leads (V7‐V9) were not analyzed in this study.
- Thirdly, it is difficult to identify the single culprit vessel in NSTEMI patients with multi‐vessel disease, although the number of these cases was small.

Furthermore, both STV5 + STV6 ≥ 2.5 mm and T‐wave imbalance had limited sensitivity in predicting the occlusive culprit LCX. It is unclear how these indicators could influence clinical decision‐making. Finally, while we attempted to remove as many secondary sources of ST segment and T wave variations as feasible in this investigation, it is unclear how the results might be used in real clinical circumstances. Emerging technology breakthroughs like computerized leads and machine learning will be able to detect an acute blocked culprit artery presenting as NSTEM more sensitively and precisely in the not‐too‐distant future.

## 6 Conclusion

In individuals with NSTEMI, the most prevalent culprit vessel with acute complete blockage is the LCX. Although patients with culprit LCX had greater troponin levels, more severe MR, and worse rates of effective reperfusion during hospitalization than patients with culprit LAD or RCA, there were equal occurrences of MACES at the one‐year follow‐up. This subset of patients may be predicted by STV5 + STV6 ≥ 2.5 mm and T‐wave imbalance. However, the sensitivity is poor, and larger sample size investigations are required to corroborate our findings.

## Author Contributions

Y.W. and F.J. designed the research.Y.W., D.P., J.D., and L.M. collected the data.Y.W. analyzed the data and prepared the manuscript. B.R.S. checked the manuscript. F.J. reviewed the manuscript. All authors read and approved the manuscript.

## Conflicts of Interest

The authors declare no conflicts of interest.

## Supporting information


Data S1.

## Data Availability

The data that support the findings of this study are not publicly available due to privacy or ethical restrictions, but may be available from the corresponding author on reasonable request.
