# Supplementary material for: Clinical and Electrocardiographic Characteristics in NSTEMI Patients With Acute Total Occlusion of Culprit Left Circumflex Artery
Source: Ann Noninvasive Electrocardiol. 2025 Apr 3;30(3):e70070. doi: 10.1111/anec.70070 (PMC11966646; doi:10.1111/anec.70070)
Supplement: Supplementary file 1 — Data S1. [file ANEC-30-e70070-s001.docx]

Supplemented Table Univariate logistic regression analysis of ECG predictors for culprit LCX.

| **Variable** | **B** | **Wald** | **P** | **OR** | **95%CI** |
| --- | --- | --- | --- | --- | --- |
| Sinus rhythm | -0.094 | 0.019 | 0.891 | 0.910 | 0.236~3.506 |
| Heart rate,bpm | -0.012 | 2.066 | 0.151 | 0.988 | 0.971~1.005 |
| PR interval,ms | 0.012 | 3.941 | **0.047** | 1.012 | 1.000~1.025 |
| QRS duration,ms | 0.013 | 1.014 | 0.314 | 1.013 | 0.988~1.039 |
| QTc interval,ms | 0.005 | 1.636 | 0.201 | 1.005 | 0.997~1.013 |
| Anterior Q-waves or QS waves | 0.094 | 0.019 | 0.891 | 1.099 | 0.285~4.233 |
| Lateral Q-waves or QS waves | 1.860 | 5.753 | **0.016** | 6.422 | 1.405~29.351 |
| Inferior Q-waves or QS waves | -0.407 | 0.997 | 0.318 | 0.666 | 0.299~1.480 |
| Prominent R-wave in V1 | 1.876 | 3.018 | 0.082 | 6.528 | 0.786~54.198 |
| Poor R-wave progression in V1-V3 | -0.017 | 0.001 | 0.973 | 0.983 | 0.361~2.674 |
| ST depression in |  |  |  |  |  |
| I、aVL | 0.711 | 2.155 | 0.142 | 2.037 | 0.788~5.266 |
| II、III、aVF | -0.482 | 1.430 | 0.232 | 0.617 | 0.280~1.361 |
| V1-V6 | 0.296 | 0.291 | 0.590 | 1.345 | 0.458~3.949 |

Supplemented Table Uni variate logistic regression analysis of ECG predictors for culprit LCX (continued).

| **Variable** | **B** | **Wald** | **P** | **OR** | **95%CI** |
| --- | --- | --- | --- | --- | --- |
| V1-V4 | 0.565 | 1.469 | 0.225 | 1.759 | 0.706~4.385 |
| V5-V6 | 0.831 | 7.162 | **0.007** | 2.295 | 1.249~4.216 |
| ST_V5_+ST_V6_ ≥2.5mm | 1.038 | 10.027 | **0.002** | 2.824 | 1.485~5.368 |
| T-wave low-flat or inversion in |  |  |  |  |  |
| I、aVL | 0.849 | 7.607 | **0.006** | 2.338 | 1.279~4.274 |
| II、III、aVF | -0.047 | 0.022 | 0.882 | 0.954 | 0.511~1.779 |
| V1-V6 | -1.438 | 5.875 | **0.015** | 0.237 | 0.074~0.759 |
| V1-V4 | -1.507 | 10.525 | **0.001** | 0.222 | 0.089~0.551 |
| V5-V6 | 0.777 | 6.327 | **0.012** | 2.174 | 1.187~3.981 |
| T-wave imbalance | 1.656 | 20.668 | **0.000** | 5.239 | 2.565~10.698 |
| de-Winter ST/T-wave complex | -0.138 | 0.028 | 0.868 | 0.871 | 0.171~4.436 |
| T2-T6≥6mm | 0.858 | 7.879 | **0.005** | 2.359 | 1.296~4.295 |
| TLI+T6≤0mm | 0.677 | 4.702 | **0.030** | 1.968 | 1.067~3.630 |
| N-wave | 1.517 | 1.883 | 0.170 | 4.560 | 0.522~39.844 |

*ST_V5_+ST_V6_ ≥2.5mm,the sum of ST amplitude of V5 and V6 ≥2.5mm;T2-T6≥6mm,the difference of T-wave amplitude between V2 and V6 ≥6mm;TL1+T6≤0mm,the sum of T-wave amplitude of L1 and V6 ≤ 0mm.Significant P values are in boldface.*
